# Supplementary material for: A Deep Insight into the Diversity of Microfungal Communities in Arctic and Antarctic Lakes
Source: J Fungi (Basel). 2023 Nov 9;9(11):1095. doi: 10.3390/jof9111095 (PMC10672340; doi:10.3390/jof9111095)
Supplement: Supplementary file 1 [file jof-09-01095-s001.zip › Table S1.pdf]

Table S1. Concentration and quality of DNA extracted from water and sediment samples.

Sample Name	Nucleic Acid Conc(ng/μL)	OD260/280	OD260/230
L1-s	92.7	2.05	1.2
L2-s	18.31	2.22	1.4
L3-s	16	1.95	1.7
L4-s	68.76	1.81	1.3
L5-s	50.97	1.75	2.04
LA-s	12.24	1.91	1.07
LB-s	25.39	1.71	1.7
LS-s	28.13	2.25	1.25
LT-s	15.81	1.84	2.41
LZ-s	14	1.98	1.21
L1-w	22.59	1.99	1.25
L2-w	27.73	2.05	2.06
L3-w	24.34	2.16	1.9
L4-w	42.65	2.04	1.52
L5-w	20	2	2.33
LA-w	24.45	2.13	1.25
LB-w	7.19	2.73	2.04
LS-w	13.25	2.54	2.06
LT-w	12.64	2.16	2.03
LZ-w	37.27	1.85	1.66
